# A Standardized Clinical Data Harmonization Pipeline for Scalable AI Application Deployment (FHIR-DHP): Validation and Usability Study

**DOI:** 10.2196/43847

**Published:** 2023-03-21

**Authors:** Elena Williams, Manuel Kienast, Evelyn Medawar, Janis Reinelt, Alberto Merola, Sophie Anne Ines Klopfenstein, Anne Rike Flint, Patrick Heeren, Akira-Sebastian Poncette, Felix Balzer, Julian Beimes, Paul von Bünau, Jonas Chromik, Bert Arnrich, Nico Scherf, Sebastian Niehaus

**Affiliations:** 1 AICURA Medical GmbH Berlin Germany; 2 Institute of Medical Informatics Charité – Universitätsmedizin Berlin Berlin Germany; 3 idalab GmbH Berlin Germany; 4 Digital Health – Connected Healthcare, Hasso Plattner Institute University of Potsdam Potsdam Germany; 5 Max Planck Institute for Human Cognitive and Brain Sciences Leipzig Germany

**Keywords:** data interoperability, fast healthcare interoperability resources, FHIR, data standardization pipeline, medical information mart for intensive care, MIMIC IV, artificial intelligence, AI application, AI, deployment, data, usability, care unit, diagnosis, cooperation, patient care, care, medical research

## Abstract

**Background:**

Increasing digitalization in the medical domain gives rise to large amounts of health care data, which has the potential to expand clinical knowledge and transform patient care if leveraged through artificial intelligence (AI). Yet, big data and AI oftentimes cannot unlock their full potential at scale, owing to nonstandardized data formats, lack of technical and semantic data interoperability, and limited cooperation between stakeholders in the health care system. Despite the existence of standardized data formats for the medical domain, such as Fast Healthcare Interoperability Resources (FHIR), their prevalence and usability for AI remain limited.

**Objective:**

In this paper, we developed a data harmonization pipeline (DHP) for clinical data sets relying on the common FHIR data standard.

**Methods:**

We validated the performance and usability of our FHIR-DHP with data from the Medical Information Mart for Intensive Care IV database.

**Results:**

We present the FHIR-DHP workflow in respect of the transformation of “raw” hospital records into a harmonized, AI-friendly data representation. The pipeline consists of the following 5 key preprocessing steps: querying of data from hospital database, FHIR mapping, syntactic validation, transfer of harmonized data into the patient-model database, and export of data in an AI-friendly format for further medical applications. A detailed example of FHIR-DHP execution was presented for clinical diagnoses records.

**Conclusions:**

Our approach enables the scalable and needs-driven data modeling of large and heterogenous clinical data sets. The FHIR-DHP is a pivotal step toward increasing cooperation, interoperability, and quality of patient care in the clinical routine and for medical research.

## Introduction

The increasing digitalization of health care creates vast amounts of clinical data that are collected and stored in an Electronic Health Record (EHR). Patient information from all medical domains is captured in diverse sets of data recorded in stand-alone systems. With the prevalent use of EHRs in health care organizations, there is abundant opportunity for the additional application of EHR data in clinical and translational research. For instance, such data can be used to develop artificial intelligence (AI) algorithms, which have the potential to transform patient care and medical research. Resource-intensive and inefficient clinical workflows could be optimized by the analysis of historical data with AI applications [1,2]. In particular, the time-consuming and financially costly process of identifying and enrolling the right patients into a clinical trial manually can be reduced significantly by automation [3,4]. However, the exchange of medical data remains limited due to the lack of data interoperability between health care providers, owing to outdated IT infrastructure, inconsistencies in data formats, poor data quality, inadequate data exchange solutions, and data silos [5,6]. To achieve data interoperability, the following steps must be incorporated: (1) integration of isolated data silos, (2) safe exchange of data, and (3) effective use of the available data [7]. Each of these operations includes database schema matching [8] and schema mapping [9], which allow translation of the relationships between the source database and the target data standard.

Employing a harmonized data format will facilitate the exchange of medical data, enabling wide-ranging data-driven collaborations within the private and public health care sectors. Data interoperability requires EHR data to be structured in a common format and in standardized terminologies. Standardization is often performed by adopting the Health Level 7 Fast Healthcare Interoperability Resources (FHIR) model [10], which is supported by numerous health care institutions and vendors of clinical information systems [11]. FHIR is an international industry standard that integrates diverse sets of data in well-defined exchangeable segments of information, which are known as FHIR resources. Therefore, FHIR facilitates interoperability between health care organizations and allows third-party developers to provide medical applications that can be easily integrated into the existing systems. FHIR enables the harmonization of data and thus allows standardized data processing as well as the rollout of AI applications across different clinics and hospitals regardless of which information system they use. Consequently, FHIR forms an important component for the scalable development and deployment of AI in clinics and hospitals.

However, to apply AI, the input data need to be adapted to the AI algorithms. The conventional AI frameworks such as Tensorflow [12] and Pytorch [13] require data to take a tensor form, which is a vector or matrix of n-dimensions that represents various types of data (eg, tabular, time series, image, and text). Since the FHIR format has a multilayered nested structure, a use case–specific data preprocessing is needed. For instance, depending on the AI application and the chosen data source, a custom data preprocessing pipeline should be designed leading to diminished AI scalability. Prior research addressed this problem in different forms but focused on individual applications, thereby constraining the purpose of FHIR to be applicable regardless of the use case [11]. There have been a few attempts to flatten the hierarchical FHIR structure and transform it into NDJSON-based data format [14] or tabular format saved in CSV files [15]. Such formats are more AI-friendly as they represent the data in a more accessible and standardized form for an application of common AI frameworks. Nonetheless, the NDJSON-based FHIR data transformation approach [14] does not provide data selection criteria and filtering capabilities [16]. The approach presented in [15] requires expert knowledge of FHIRPath query language. Moreover, FHIR-based data preprocessing pipelines have been implemented in different contexts, for instance, as electronic data capture [17], as a natural language processing tool [12], and as a standardization protocol based on the Resource Description Framework [6]. Despite the immense benefit they offer regarding processing EHR data, existing approaches are limited to specific use cases or require considerable data preparation to perform standardization. Furthermore, their final output is not easily accessible by common data preprocessing tools and thus hinders the application of AI.

In this paper, we address the challenge of data interoperability in the health care sector by proposing an FHIR data harmonization pipeline (DHP) that provides EHR data in an AI-friendly format. The newly developed FHIR-DHP represents a data workflow solution that includes the aforementioned operations, such as data exchange, mapping, and export. Data privacy is a delicate topic in health care and is of great ethical concern [18]. Given the degree of automation, FHIR-DHP should allow the preprocessing of unseen data in an isolated hospital environment, which makes harmonization privacy preserving.

## Methods

### Ethical Considerations

The authors did not seek an ethics review board assessment due to the methodology of the study, which included open datasets and data preprocessing pipelines only.

### FHIR-DHP Architecture Development

In our work, we propose a generic solution to harmonize hospital EHR data. The FHIR-DHP was designed based on the extract-transform-load framework [19], in which the data are pulled out (ie, queried) from diverse sources, processed into the desired format, and loaded into a data warehouse, namely the ”patient-model” database (DB). As the hospital database contains highly sensitive patient data, it is located behind the hospital’s security infrastructure and is completely isolated from outside access. Therefore, an edge-computation solution was designed, bringing the FHIR-DHP into the hospital’s own infrastructure. The edge-computation solution represents a set of frameworks that perform data querying, preprocessing, storage, and export. In this setting, direct access to the sensitive data is not required to run the standardization pipeline. The queries to the data are defined beforehand based on the database documentation.

To bring the data into a harmonized form, we used an FHIR data model, which is applied by mapping the relationships between the source database and the desired data standard. The FHIR standard is straightforward to implement because it provides a choice of JSON, XML, or resource description format for data representation. The mapping pipeline was developed in the Python programming language to translate queried hospital data into matching FHIR concepts and save the resulting resources in JSON format. The semantics of features from the source database and FHIR concepts are examined using available database and FHIR documentation. The conversion to FHIR was designed to only support a core release 4 standard of the FHIR format to allow generic data preprocessing.

To prevent errors in the remote data standardization scenario, the syntactic validation of FHIR resources is necessary. For instance, the conversion of data types can sometimes lead to erroneous values, especially with date features. Automatic syntactic validation allows the logging of occurred errors and the improvement of harmonization pipeline when working with unseen data. When syntactic validation is completed, FHIR resources should be transferred to the data warehouse to allow the fast and easy retrieval of standardized data for AI applications.

In the final stage of data export, we designed the output that provides the benefits of the original FHIR format with a high level of clinical detail that is also easily accessible for computational tools. We wanted to restructure the data representation in a way that supports effortless data selection and filtering capabilities and would not require a knowledge of FHIRPath query language. Consequently, this output format would enable the smooth conversion of data into a “tensor” format required by conventional AI frameworks.

### FHIR-DHP Validation

To demonstrate and evaluate how the FHIR-DHP works, we used the openly available Medical Information Mart for Intensive Care IV (MIMIC IV) database [20]. MIMIC IV includes patient data from the intensive care units at a tertiary academic medical center in Boston, MA, United States. We selected a wide range of tables from MIMIC IV, which cover most of the events occurring during the hospital stay as well as core patient details, information about admissions, and hospital transfers (further referred to as core tables). The event tables include laboratory results, diagnoses, prescriptions, and other details, as shown in Table 1. In addition, MIMIC IV includes the so-called reference tables containing matching dictionaries with medical terms that are used in the hospital records.

**Table 1:** Selected core and event Medical Information Mart for Intensive Care IV (MIMIC IV) tables as well as the reference dictionary tables that were merged together with core and event tables for Fast Healthcare Interoperability Resources mapping.

Selected core and event MIMIC IV tables	Selected MIMIC IV reference tables
Patient	—^a^
Admissions	—
Transfers	—
Chartevents	d_items
Labevents	d_labitems
Procedureevents	d_items
Prescriptions	—
Inputevents	d_items
Microbiologyevents	—
Outputevents	d_items
Procedures_icd	d_icd_procedures
Diagnoses_icd	d_icd_diagnoses

^a^Not available.

The selected tables were mapped to FHIR standard. Automatic semantic validation is unfeasible, so 2 of the authors manually validated the mapping semantics independently of each other. There are many tools that perform automatic syntactic validation, such as the Python-based package “fhir.resources” used herein [21]. To evaluate the exporting of data from the patient-model DB, we retrieved the diagnosis records.

## Results

### FHIR-DHP Architecture

The approach presented here represents a scalable protocol for harmonizing hospital EHR data sets based on 5 stages from data query to data export in a standardized format.

#### Querying Data From the Hospital Database

To connect the FHIR-DHP pipeline to the hospital DB, a communication server is employed. This server runs all necessary queries to retrieve the patient data. The query execution can be run at regular intervals as well as in batches of patients, so as not to overload the data pipeline. Furthermore, the queries prestructure the data according to their semantic relations before proceeding to data mapping.

#### Mapping Data to FHIR

FHIR allows describing data formats and elements that are recorded as “resources” and an application programming interface for exchanging EHRs. To perform the mappings, semantics of features from the source database and FHIR concepts are explored as well as the relationships between the data tables. Consequently, the mappings between the database tables and FHIR resources are defined. Features where a matching FHIR concept is not found are excluded. The resulting FHIR resources are then saved in JSON format.

#### Syntactic Validation of FHIR Mappings

During validation, mapped data are ensured to have the correct data types as well as the syntactic format where the hierarchy is maintained, and entries follow FHIR standard specifications. All mappings are validated first during the development stage to identify structural errors and data type inconsistencies. A validation algorithm is incorporated into the pipeline to confirm the correctness of the transformed data in the remote data standardization scenario.

#### Transferring FHIR Resources to Patient-Model DB

The DB of choice for the patient model is Postgres [22], which is an open-source relational DB management system featuring SQL compliance and storage of JSON documents. The database for the FHIR resources is used to harmonize the locally available data only once to allow the further application of various medical AI-based solutions. The data are stored according to FHIR resource type where each resource is saved in a separate JSON structure.

#### Exporting Data Into Custom JSON format

To export the data from the patient-model DB, the selection is performed by outlining the tables and features of interest in a configuration file, which is then used to determine which harmonized data should be queried. FHIRPath queries were written to retrieve all elements from FHIR resources adhering to specific formatting rules in respect of the predefined key-value structure and to place the extracted elements into the custom JSON file. Such transformation flattens the hierarchical structure of FHIR resources and makes the data more accessible for common data preprocessing tools. The final flattened output does not require expert knowledge of FHIRPath query language and supports effortless data selection and filtering. The resulting file also allows the uncomplicated conversion of data into a “tensor” format required by conventional AI frameworks and fast data selection based on the following 4 keys: feature_name, table_name, value, and metadata.

**Figure 1:**
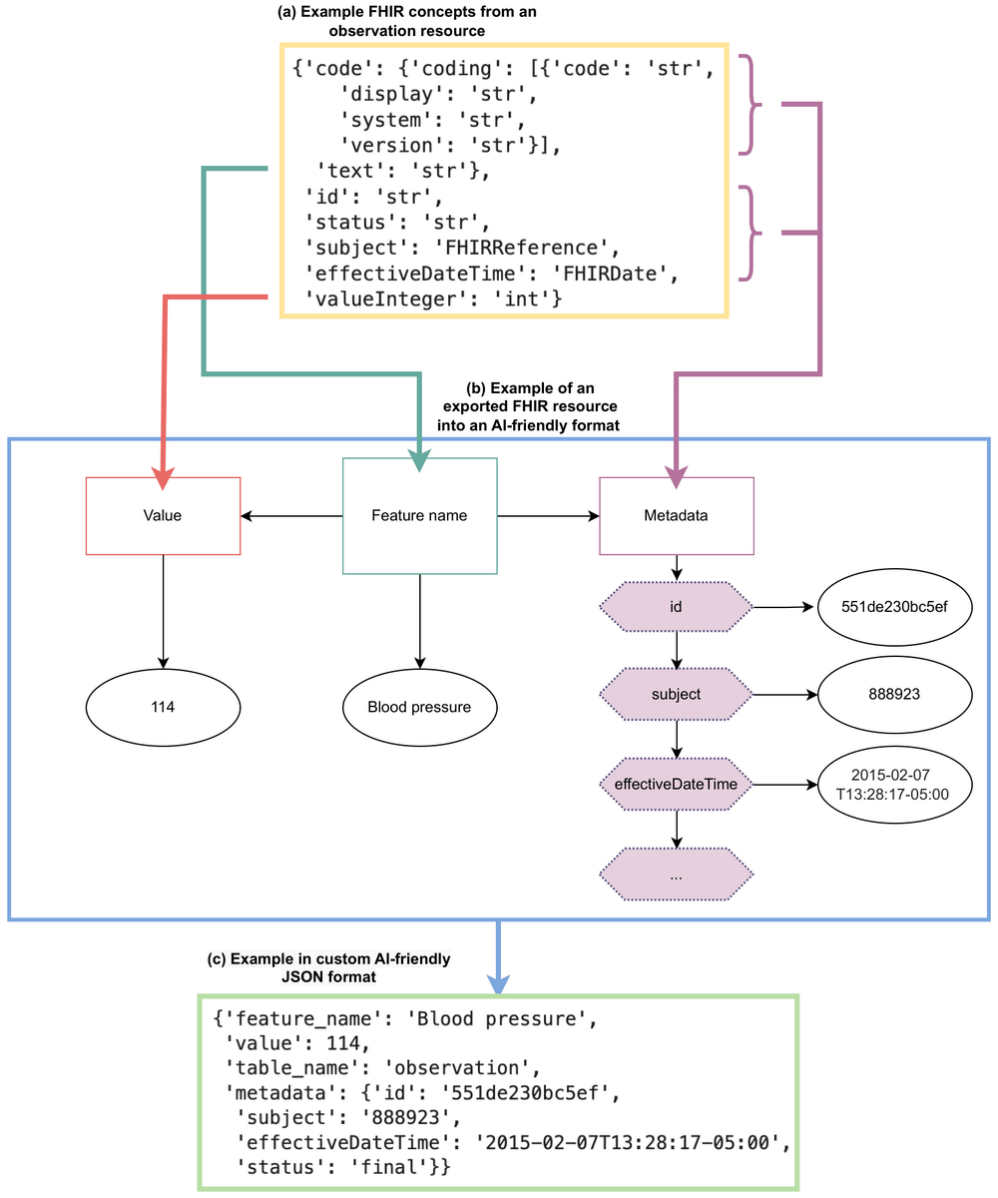
Conceptual overview for an exemplary Fast Healthcare Interoperability Resources (FHIR) structure and hospital record, which are transformed from FHIR standard to an artificial intelligence (AI)–friendly format.

In Figure 1, we demonstrate how the FHIR-DHP recodes nested FHIR syntax to more accessible features in an AI-friendly format. Example FHIR concepts from an observation resource are given in Figure 1a, where the code’s entity “text” defines the record or measurement label. The entity “text” is often duplicated in the item “display.” However, depending on the coding system, this “display” item can change, whereas “text” always stays the same and is therefore used as a feature name. The information from the FHIR resource is grouped into the 4 concept keys of feature name (eg, “Blood pressure”), value (eg, “114”), table name (eg, “observation”), and metadata (Figure 1b). For a given FHIR resource type, the metadata may include concepts such as dates, references, coding system details, and resource ID, among other things. As an output, feature names together with a corresponding value and available metadata are provided in a custom JSON structure (Figure 1c). The defined format allows uncomplicated data selection and aggregation based on resource type (eg, “table_name”), feature name, and value. Additional information in a standardized format can be easily accessed from the metadata key and allows further data manipulation.

### FHIR-DHP Validation

The MIMIC IV data were queried accordingly to the defined FHIR mappings. The core and event MIMIC IV tables were merged with reference tables to contain a complete description of the hospital records. As a result, the data were grouped and restructured into the information blocks required in FHIR standard. Manual independent validation of the mapping semantics resulted in slight discrepancies, which were subsequently resolved to adhere closely to the FHIR standard. The automatic syntactic validation allowed the prompt verification of standardization operations.

Table 2 shows to which FHIR resources the MIMIC IV tables were mapped. The largest proportion of tables (4 out of 12 tables) were mapped to the *Observation* FHIR resource type, which included lab, microbiology, output, and charted events collected throughout the patient’s stay. The information on admissions and transfers was translated into the *Encounter* FHIR resource (2 out of 12 tables). Procedure events and International Classification of Diseases codes (2 out of 12 tables) were stored in the *Procedure* FHIR resource. Given that the prescriptions table contains medication requests (1 out of 12 tables) and the input events table holds records of medication administration (1 out of 12 tables), these tables were mapped to the corresponding FHIR resource types. Finally, the *Condition* FHIR resource was used to map the table with the patients’ diagnosis details (1 out of 12 tables).

In Table 3, we demonstrate how the mapping of the MIMIC IV “diagnoses_icd” table to *Condition* FHIR resource was conducted. Multiple columns of the “diagnoses_icd” table such as “icd_code”, “icd_version,” and “long_title” were mapped to the FHIR “condition.code” concept, which has a nested structure and provides keys to store the exact International Classification of Diseases code, the version of the coding system, and the code title. The full diagnosis title was mapped both to the “display” and “text” entities.

**Figure 2:**
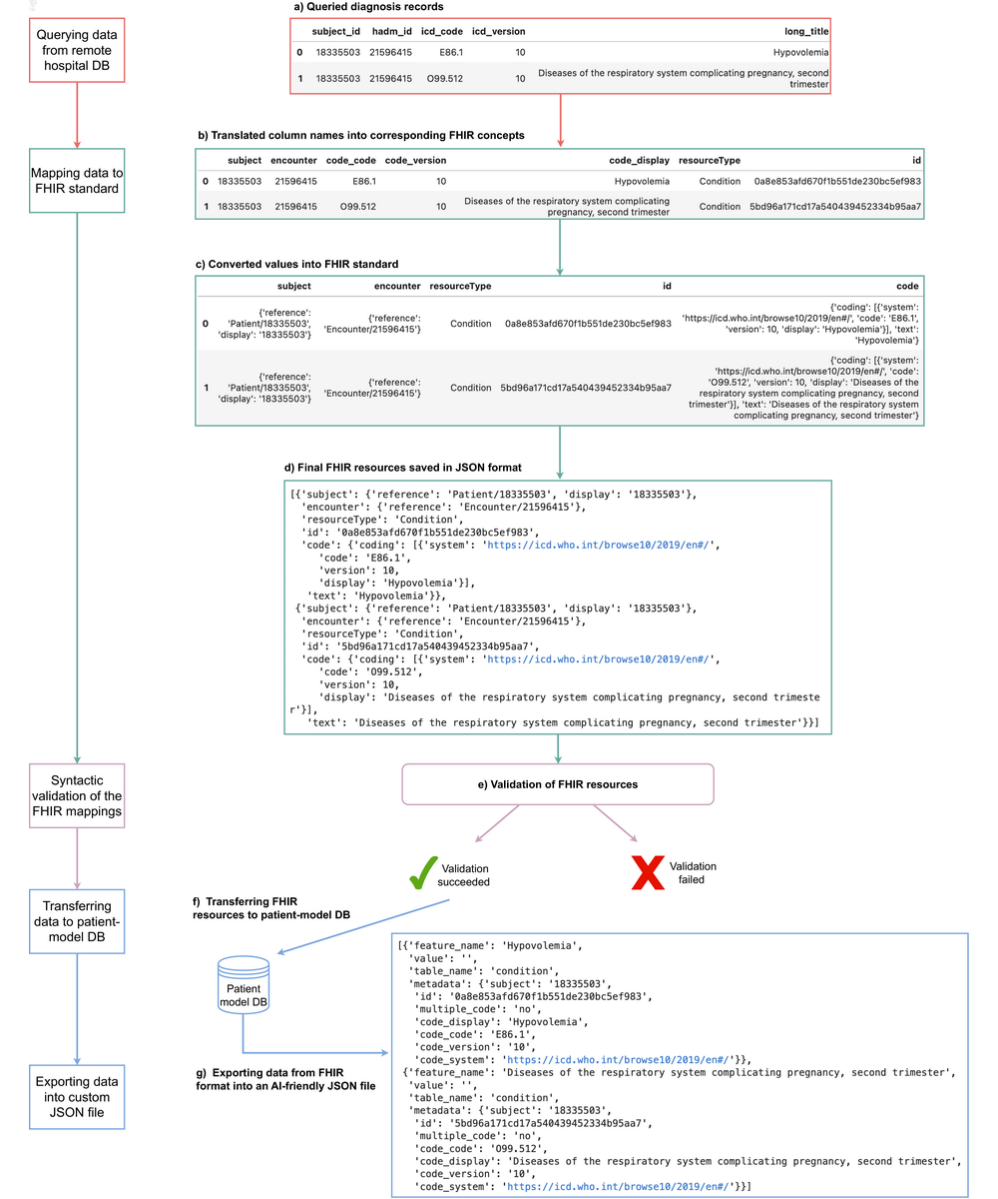
Flowchart showing an example diagnosis data being processed through the 5 stages in Fast Healthcare Interoperability Resources (FHIR) data harmonization pipeline (DHP). The first stage (a) includes querying of the diagnoses records, at the second stage (b-c) the data are mapped to FHIR standard, and the third stage carries out the syntactic resource validation. (f) If the FHIR resource is successfully validated, it is being transferred into the patient-model database (DB), and then (g) exported in a custom artificial intelligence (AI)–friendly JSON format.

Figure 2 shows an example of how queried diagnoses records are harmonized to an AI-friendly format. The standardization follows the FHIR-DHP stages described above. At first, the raw data from tables “diagnoses_icd” and “d_icd_diagnoses” are queried (Figure 2a) and merged accordingly to the defined FHIR mappings. Then, the features are renamed as defined in Table 3 for the FHIR condition resource, and the required entities such as “resourceType” and “id” are created (Figure 2b). Finally, the values are placed into a nested FHIR structure (Figure 2c), and subsequently, the data are transformed into a JSON format (Figure 2d), which can be automatically validated (Figure 2e) and saved in the patient-model DB. When the resource is not approved in terms of its syntactic quality (eg, data type, nested structure, or cardinality), an error is raised, which prevents the further saving of this resource in the patient-model DB (Figure 2e). Otherwise, the resource is transferred into a storage (Figure 2f), and the requested data are exported in a custom AI-friendly JSON format (Figure 2g).

We provide an example of a further 2-step transformation of harmonized diagnosis data to a “tensor” format in Multimedia Appendix 1 [12,23].

**Table 2:** Overview of the mappings performed on the selected Medical Information Mart for Intensive Care (MIMIC) database (DB) tables to Fast Healthcare Interoperability Resources (FHIR) types.

MIMIC IV DB	FHIR resource type
Patients	Patient
Admissions	Encounter
Transfers	Encounter
Chartevents	Observation
Labevents	Observation
Procedureevents	Procedure
Prescriptions	MedicationRequest
Inputevents	MedicationAdministration
Microbiologyevents	Observation
Outputevents	Observation
Procedure_icd	Procedure
Diagnoses_icd	Condition

**Table 3:** Mapping of “diagnoses_icd” table to Condition Fast Healthcare Interoperability Resources (FHIR) resource.

MIMIC^a^ format	FHIR resource format
mimic.diagnoses_icd.subject_id	fhir.condition.subject
mimic.diagnoses_icd.hadm_id	fhir.condition.encounter
mimic.diagnoses_icd.icd_code	fhir.condition.code_code
mimic.diagnoses_icd.icd_version	fhir.condition.code_version
mimic.diagnoses_icd.long_title	fhir.condition.code_display
mimic.diagnoses_icd.long_title	fhir.condition.code_text

^a^MIMIC: Medical Information Mart for Intensive Care.

## Discussion

### Principal Findings

The Harmonization of EHR data is a crucial step toward increasing cooperation, interoperability, and quality of patient care in the clinical routine and medical research. To drive the harmonization of medical data forward, we developed the FHIR-DHP and evaluated it on key MIMIC IV tables. A detailed example of data standardization was presented for clinical diagnosis records from the MIMIC IV database. The FHIR-DHP allows the querying of health data in an isolated environment by employing an edge-computation solution and a communication server, which retrieve patient data and prestructure it for further mapping to the FHIR standard. A validation step ensures syntactic compliance and initiates the transfer of formatted data to the patient-model DB. The data export provides FHIR resources in a custom JSON file format.

Owing to the FHIR format’s multilayered nested structure, its accessibility for AI algorithms is low as it requires transformation into a format compatible with common data preprocessing tools. Thus far, a number of studies have attempted to solve this problem. However, the final output of these studies has not supported data selection criteria and filtering capabilities [14] and requires expert knowledge of FHIRPath query language [15]. In this study, we introduce a custom JSON format that represents a higher level of abstraction to support easier data selection based on the following 4 keys: feature_name, table_name, value, and metadata. Moreover, the newly developed JSON structure fits the expected data format of common data preprocessing frameworks, which are designed to work efficiently with tabular data. As a result, the output presented facilitates the generic and fast deployment of AI and patient cohort identification algorithms.

In comparison to [17,24], the details of FHIR-DHP execution inside the hospital environment in respect of protecting data privacy are discussed. This step, though crucial, is often omitted and left out of the published standardization protocols. The edge-computation solution sets up the FHIR-DHP in a privacy-preserving way where the preprocessing of the patient-related data is performed inside the hospital and is completely isolated from outside access. The so-called federated learning (FL) framework [25] can be integrated into the FHIR-DHP workflow to run algorithms locally, using data from the on-premises database in the respective hospitals and to merge model parameters centrally in the cloud without any patient data leaving the hospital. The FL framework requires data to be in a consistent format across various hospital systems. The developed pipeline achieves such a format and enables the scaling of AI applications.

Thus far, there are only 2 studies attempting to perform the mapping of an MIMIC IV database [26,27]. In [26], the mapping was performed on fewer tables than our approach (8 versus 12 tables). The FHIR mappings from [27] have been recently released and were not yet widely validated. Similar to the approach taken in [17,24,26], FHIR-DHP includes the verification of the performed FHIR mapping, which is essential to ensure the validity of data transformation and to adhere to FHIR version updates. Moreover, in comparison to [17,24,26], FHIR-DHP represents a generic approach to standardize EHR data and can be applied to various hospital database systems.

With the introduction of the FHIR-DHP into the hospital environment, a number of patient-stay parameters can be potentially optimized using AI-based algorithms. For example, the length of stay as well as mortality could be reduced [28], and patients suitable for trial treatment could be automatically and efficiently identified [29]. In consequence, the financial impact on medical providers in respect of personnel time and resources would decrease considerably. The FHIR-DHP aims to bring health care closer to digital transformation and thus toward “Healthcare 4.0” [30] by making EHR data usable “from bedside-to-bench.” By inverting the idea of translational research, in contrast to “from bench-to-bedside,” accessing the full potential of medical big data with AI will further inform and advance basic research.

### Limitations

There are several limitations that we would like to emphasize. FHIR-DHP only works with a core standard of the FHIR format. Those core FHIR resource types have a bounded set of concepts that present a constraint to mapping accuracy. Although the standard resources can be expanded using a profiling technique or FHIR extensions, the use of those would make the FHIR-DHP less generic. Hence, we implemented the mapping using only the standard FHIR resources and omitted some of the MIMIC IV data features that did not have a matching concept in FHIR. Additionally, the FHIR mapping step is subject to the extent of the detail of the database documentation used to infer the semantic and syntactic properties of the data. A solution for an automatic concept recognition can potentially solve this problem. The existing approach in [6] is limited to a small number of FHIR resources and requires an extensive data preparation. Further experiments in this direction could alleviate the concept-matching problem and the requirement for a detailed database description. Moreover, the validation and robustness of FHIR-DHP needs to be tested on other EHR data sets to evaluate its generic setup. In addition, to validate the FHIR-DHP compatibility with machine learning pipelines, further experiments are needed.

### Future Prospects

The proposed FHIR-DHP pipeline highlights the therein featured essential data standardization stages and holds the potential to becoming an interoperable harmonization system with an AI-friendly data format. FHIR-DHP enables interoperability and cooperation between clinical institutions and a rapid patient cohort identification for clinical trials; it also unlocks the potential of big medical data.

### Conclusions

We provide a comprehensive approach to transforming unstandardized EHR data into a harmonized multilayered nested FHIR format and then to a more readable and more efficient AI-friendly JSON structure. We developed a 5-stage data harmonization pipeline, which includes validation checks. The AI-friendly format of hospital data allows the generic and fast integration of both AI and patient cohort identification algorithms. Harmonized and standardized health care data are of great value to advancing efficiency in big data processing, cooperation, and multicenter data exchange in the clinical sector, boosting medical research, patient care, and clinical trial cohort identification. The next steps would include validating our approach in a hospital environment and applying a privacy-preserving FL framework to make use of advanced AI deployment.

## Appendix

Multimedia Appendix 1 Transformation of data saved in custom JSON to tensor format.

## Abbreviations

AI: artificial intelligence
DB: database
DHP: data harmonization pipeline
EHR: Electronic Health Record
FHIR: Fast Healthcare Interoperability Resources
FL: federated learning
MIMIC: Medical Information Mart for Intensive Care

## Availability of Data and Materials

The MIMIC IV database used in this study is openly available to credentialed users who sign the “Data Use Agreement” at PhysioNet website [20]. The code is not publicly available due to privacy, but a demo is available from the corresponding author on request.
