# Supplementary material for: A Standardized Clinical Data Harmonization Pipeline for Scalable AI Application Deployment (FHIR-DHP): Validation and Usability Study
Source: JMIR Med Inform. 2023 Mar 21;11:e43847. doi: 10.2196/43847 (PMC10131740; doi:10.2196/43847)
Supplement: Multimedia Appendix 1 [file medinform_v11i1e43847_app1.docx]

**Multimedia Appendix 1 – Transformation of data saved in custom JSON to tensor format**

Figure 1. Flow chart showing an example diagnoses data saved in proposed custom JSON file being transformed to a “tensor” format. The diagnoses records saved in custom JSON (a) are first flattened to a tabular form (b), and then transformed into “tensor” (c).

In this section we would like to demonstrate how the proposed output custom JSON format can be easily transformed into an input data for common AI frameworks. To achieve a “tensor” shape we applied a two-step transformation. As shown in **Figure 1a**, an example diagnoses records are exported a custom AI-friendly format. In the first transformation step (see **Figure 1b**) the JSON format is flattened into a tabular form using *json_normalize* function from Pandas data preprocessing toolkit [23]. The second step, where the tabular data is transformed into a “tensor” form, is performed using *convert_to_tensor* function from the conventional AI framework Tensorflow [12].
